# Comparing the efficacy of different intra-articular injections for knee osteoarthritis: A network analysis

**DOI:** 10.1097/MD.0000000000029655

**Published:** 2022-08-05

**Authors:** Xingzhen Lin, Fang Zhi, Qin Lan, Weixiong Deng, Xinju Hou, Qing Wan

**Affiliations:** a Nanchang Hongdu Hospital of Traditional Chinese Medicine, Nanchang, Jiangxi Province, China.

**Keywords:** hyaluronic acid, network, osteoarthritis, ozone analysis, platelet-rich plasma

## Abstract

**Background:**

The findings on the effectiveness of platelet-rich plasma, ozone, and hyaluronic acid in the treatment of osteoarthritis of the knee are controversial, and the existing original studies and meta-analyses are mostly comparisons of a single joint cavity injection method, lacking direct and indirect comparisons of different drugs in the joint cavity. The lack of direct and indirect comparisons of different drugs in the joint cavity makes it difficult to have a clearer and more comprehensive understanding of joint cavity injection methods. In this study, the efficacy of platelet-rich, ozone, sodium hyaluronate, and combined knee cavity injections were compared directly or indirectly using a reticulated meta-analysis in this field, and the efficacy of treatment measures was ranked to provide more comprehensive and reliable evidence-based clinical evidence for the selection of knee cavity injections in osteoarthritis of the knee.

**Objective:**

To compare the effects of platelet-rich plasma, ozone, and sodium glassate injection interventions on the efficacy of osteoarthritis of the knee through reticulated Meta-analysis, and to comprehensively compare the clinical effectiveness of platelet-rich plasma, ozone, and sodium glassate injection joint cavity injection for the treatment of osteoarthritis of the knee.

**Methods:**

The PubMed, CBM, CNKI, VIP, and Wan-Fang databases were searched for information on the effectiveness of platelet-rich plasma, ozone, and sodium vitrate injection for the comparative treatment of osteoarthritis of the knee, with a search time frame of each database from the date of creation to July 20, 2021. Two investigators independently screened the literature, extracted data according to inclusion and exclusion criteria, and evaluated the quality of the literature in parallel. Statistical analysis was performed using Stata 16.0 software to compare the differences in the efficacy of each treatment measure using the ratio and 95% confidence interval as effect indicators and to rank the efficacy.

**Results:**

Thirty-three RCTs with 7003 patients with osteoarthritis of the knee were included, involving 5 therapeutic measures. Meta-analysis showed that the efficacy of platelet-rich plasma injection was superior to both ozone and hyaluronic acid therapies. Hyaluronic acid+ozone and platelet-rich plasma+hyaluronic acid were both superior to ozone and hyaluronic acid monotherapy. The differences in efficacy between hyaluronic acid and ozone compared with platelet-rich plasma were statistically significant, and the differences in efficacy between the 2 combination therapies (platelet-rich plasma+hyaluronic acid, hyaluronic acid+ozone) and the 3 monotherapies (platelet-rich plasma, ozone, hyaluronic acid) were statistically significant. Platelet-rich plasma+hyaluronic acid, hyaluronic acid+ozone compared with 3 monotherapies (platelet-rich plasma, ozone, hyaluronic acid) were statistically significant, except for the difference in efficacy with platelet-rich plasma, which was not statistically significant, indicating that this platelet-rich plasma+hyaluronic acid and Hyaluronic acid+ozone combination therapy was superior to monotherapy. Also, the efficacy of platelet-rich plasma was better than hyaluronic acid and ozone and the difference was statistically significant, indicating that platelet-rich plasma was more effective than ozone and sodium glass in the treatment of osteoarthritis of the knee in monotherapy.

**Conclusion:**

It is believed that in the course of clinical practice, hyaluronic acid+ozone or platelet-rich plasma+hyaluronic acid combination therapy or platelet-rich plasma therapy can be preferred for patients with osteoarthritis of the knee.

## 1. Introduction

Knee osteoarthritis (KOA) is a chronic degenerative joint disease characterized by degeneration of articular cartilage, subchondral bone changes, and bone redundancy in middle-aged and elderly people, with pain and loss of mobility as the main clinical manifestations.^[1]^ In recent years, the incidence of KOA has been increasing year by year, and about 60% of people over 50 years of age have X-ray imaging of KOA, and about 80% of people over 65 years of age have X-ray imaging of KOA,^[2]^ which is one of the main diseases that lead to functional disability, cause economic loss and affect the social development of social groups,^[3]^ and with the advent of aging in China, the obese population has increased significantly, and according to statistics The number of people suffering from KOA is expected to be as many as 180 million in 2020,^[4]^ and KOA will become one of the leading causes of exercise and chronic disability affecting the elderly,^[5]^ which will have a huge impact on patients and society.

The current clinical treatment of KOA is divided into surgical treatment and conservative treatment. Surgical treatment is often surgically traumatic and costly and is also accompanied by the risk of surgical failure and subsequent revision, while conservative treatment options are accepted by most people because they have no obvious toxic side effects, mainly including physical factor therapy, pharmacotherapy, injection therapy, and Chinese herbal medicine, etc. Among them, knee joint cavity injection therapy is one of the common methods of conservative treatment for KOA, in which the main injectable drugs used are platelet-rich Plasma, ozone, and sodium hyaluronate.

Several studies have shown the efficacy of platelet-rich, ozone, and sodium hyaluronate knee injections in knee osteoarthritis, but their conclusions are controversial. Most of the existing original studies are single clinical efficacy analyses of platelet-rich, ozone, and sodium hyaluronate, and direct comparisons between platelet-rich, ozone, and sodium hyaluronate are lacking. This prevents researchers from comprehensively and systematically evaluating the clinical efficacy of platelet-rich, ozone, and sodium hyaluronate intra-articular injections, which is not conducive to the selection and promotion of optimal treatment regimens. In clinical work, the choice of knee joint cavity injection for the treatment of patients with Kellgren-Lawrence grade II-III osteoarthritis of the knee is mostly based on personal experience, lacking scientific basis and evidence support, and is not included in international guidelines, a status quo that is not conducive to the treatment of patients and the standardized management of medical quality. Therefore, in this study, the efficacy of platelet-rich, ozone, sodium hyaluronate, and combined knee cavity injections were directly or indirectly compared using a reticulated meta-analysis in this field, and the efficacy of treatment measures was ranked, to provide more comprehensive and reliable evidence-based medicine for the selection of knee cavity injections for the clinical treatment of osteoarthritis of the knee.

## 2. Data and Methods

The article is reported following The National Institute for Health and Care Excellence (NICE) Reticulated Meta-analysis Reporting Specification. ^[6]^

### 2.1. Inclusion and exclusion criteria

#### 2.1.1. Subjects.

Patients with osteoarthritis of the knee: ① Recurrent knee pain within the last 1 month. ② X-ray film shows narrowing of the joint space, subchondral sclerosis and/or cystic changes, and formation of bony redundancy at the joint margin. ③Joint fluid (at least 2 times) was clear with a white blood cell count <2000/mL. ④Middle-aged and elderly patients (age ≥40 years). ⑤ Morning stiffness ≤3 minutes. ⑥Bone rubbing sound during activity. All criteria must be satisfied at the same time: ① or ①③⑤⑥ or ①④⑤⑥. There was no significant baseline imbalance in age, gender, disease duration, and severity of disease in patients in different treatment measure groups of the same study, and they should be comparable.

#### 2.1.2. Interventions.

Include at least 2 of the different knee injections of platelet-rich plasma, ozone, and sodium vitrate injection. Discontinue all relevant adjuvant medications during the treatment period in addition to the studied interventions.

#### 2.1.3. Outcome indicators.

The efficiency of different knee cavity injections for the treatment of osteoarthritis of the knee. and there are clear criteria for effectiveness evaluation in the literature.

#### 2.1.4. Exclusion criteria.

①Duplicate published literature. ②Conference papers and letters. ③Studies with an unclear description of Chinese medicine or the addition of other drugs such as hormones and NSAIDs during treatment. ④Studies with incomplete or incorrect data information and fruitless contact with authors.

### 2.2. Search strategy

Computer searches were conducted for relevant clinical trials in PubMed, VIP, CBM, CNKI, and Wan-Fang databases. The English search terms mainly including “Osteoarthritis,Knee”, “Osteoarthritides, Knee”, ”Knee Osteoarthritis”, “Knee Osteoarthritides”, “Osteoarthritis Of Knee”, “Osteoarthritis,Knee”, “Osteoarthritis,Knee”, “Osteoarthritis,Knee”. “Osteoarthritis Of Knees”, “Knee OA”, “KOA”, “Platelet-rich plasma”, “Ozone”, “Sodium hyaluronate”, “HA “Osteosynthesis,knee”, “osteoarthritis of the knee”, “osteoarthritis of the knee”, “osteoarthritis of the knee”, “osteoarthritis of the knee”. “Platelet-rich plasma”, “ozone”, “sodium hyaluronate”, “vitronectin”, etc. The search was conducted using a combination of subject terms and free words, connected using the corresponding Boolean logic operators. No language restrictions were applied, and the search period was from the date of creation of each database to July 20, 2021.

### 2.3. Literature screening and data extraction

Two investigators independently screened the literature according to the inclusion and exclusion criteria, extracted the data according to the predefined data extraction form, cross-checked the data, and in case of disagreement, agreed through mutual discussion or referred to the third investigator for decision. Data extraction included basic information about the literature (literature number, title, first author, year of publication, etc.), study-related information (mean age of patients, gender composition, disease classification, diagnostic criteria, interventions, frequency of interventions, duration of treatment, follow-up time, efficacy evaluation criteria, and data on outcome indicators) and relevant elements of risk of bias evaluation.

### 2.4. Statistical analysis

Trials with 3 and more arms were first split into all possible combinations of 2 arms and evidence network plots were drawn for the comparison of each treatment measure.^[7]^ Comparison-corrected funnel plots were produced to evaluate the interventions for small sample effects or publication bias. Inconsistency factors (IF) and their 95% confidence interval (CI) were calculated to evaluate the consistency of each closure, with the lower 95% CI equal to 0 considered as good consistency, otherwise, the closure was considered to have significant inconsistency.^[8]^ Using a Bayesian Markov Chain Monte Carlo (MCMC) random-effects model, 3 chains were used for simulation, and the number of iterations was set to 50,000, with the first 20,000 used for annealing to eliminate the effect of initial values and the second 30,000 used for sampling, to calculate the ratio of the effectiveness of each treatment measure compared (odds ratio, OR) values and 95% CI with 95% CI, not including 1. *P* < .05 was considered statistically significant.^[9]^ Sensitivity analysis was performed using MCMC fixed-effects model to evaluate the stability of the study results, and the parameters were set as in the random-effects model. SUCRA graphs were plotted to predict the ranking of the efficacy of each treatment measure, with a larger area under the curve (0%–100%) indicating a better treatment measure.^[10]^ The above graphs were plotted using Stata 16.0 for statistical analysis.

## 3. Results

### 3.1. Literature search results

**Figure 1.**
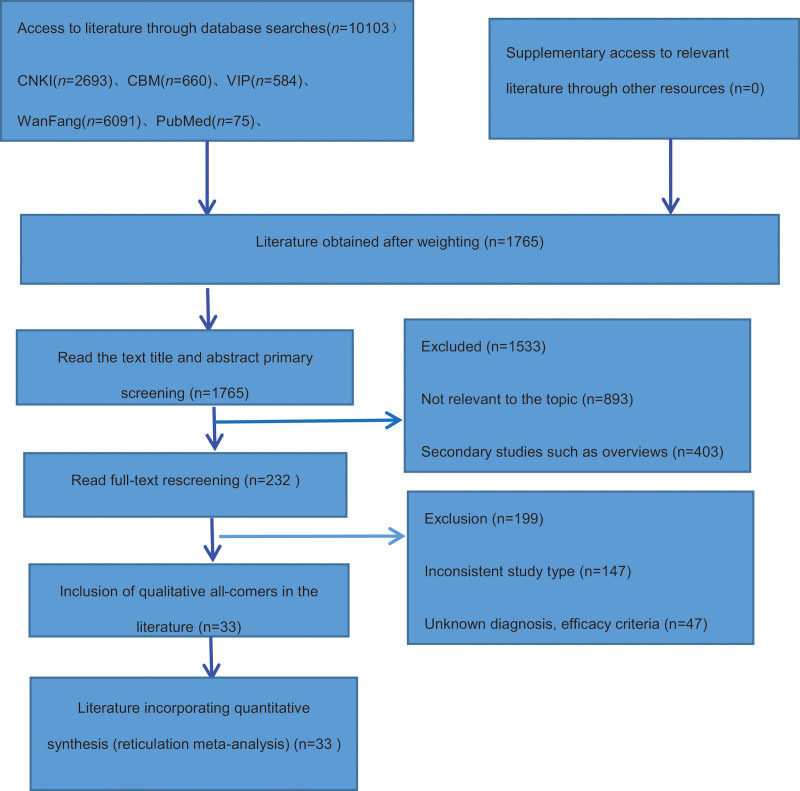
Flowchart of article screening and selection process.

After the initial screening of 10,103 papers in each database,1765 papers were obtained after ranking by NoteExpress 3.1 software, and 33 RCTs were finally included after the initial screening of titles and abstracts and re-screening by reading the full text.^[11–43]^ The flow chart of literature screening is shown in Figure 1.

### 3.2. Basic characteristics of the included literature

A total of 7003 patients with clinically confirmed osteoarthritis of the knee in 33 studies, all with a mean age >50 years, reported comparable or nonsignificant differences in age, sex, disease duration, and severity between groups, with sample sizes ranging from 12 to 126 cases. Five studies were 3-arm trials^[15,22,26,30,35]^ and the others were 2-arm trials. A total of 5 combination therapies involving platelet-rich plasma, ozone, sodium hyaluronate, and combinations between them were used (Table 1).

**Table 1:** General characteristics and quality assessment of the studies included in this network meta-analysis.

	Treatment group 1			Treatment group 2			Ending indicators
Inclusion in the study	Interventions	Number of cases (male/female)	Age (years)	Interventions	Number of cases (male/female)	Age (years)	
Zhang Lei 2019	PRP+HA	42 (18/24)	56.28 ± 4.36	HA	42(16/26)	57.13 ± 4.89	①+②
Jiang Li 2016	HA	43(27/16)	65.12 ± 3.12	HA+OZ	43(29/14)	65.83 ± 3.59	②+①
Li Jun 2020	PRP	45(25/20)	52.09 ± 2.13	HA	43(27/18)	53.24 ± 2.17	②+③
Sugar Zi Peng 2020	PRP	50(22/28)	63.49 ± 3.32	HA	50(23/27)	64.27 ± 3.54	③
Liao Dehua 2020	HA	43(19/24)	58.3 ± 4.6	OZ	44(21/23)	59.1 ± 4.7	②+⑩
				HA+OZ	43(20/23)	58.7 ± 4.3	
Wu Chunxi 2015	HA+OZ	118		HA	118		②
Li Bao 2020	HA+OZ	73(19/54)	57.2 ± 8.4	HA	73(22/51)	59.6 ± 8.4	②+⑧
Chen Ping 2019	OZ	54(20/34)	58.6 ± 3.4	HA	54(22/32)	58.5 ± 3.4	②
Kong Deguang 2020	HA+OZ	115(65/50)	64.3 ± 6.8	HA	115(57/58)	64.9 ± 7.1	②/①
Ji Changkun 2020	PRP	90(65/25)	60.9 ± 9.4	HA	79(45/34)	42.5 ± 9.5	③+①
Li Xiaoyang 2019	OZ	30(16/14)	66.35 ± 6.05	HA	30(14/16)	66.42 ± 6.34	②+⑩
Meng Tao 2018	OZ	46(21/25)	61.27 ± 8.31	HA	46(20/26)	61.80 ± 8.26	②+①
				HA+OZ	46(21/25)	61.58 ± 8.36	
Pan Lutao 2018	HA+OZ	64(14/50)	64.2 ± 6.4	HA	64(16/48)	63.4 ± 5.8	②+④
Bo Qimin 2020	HA+OZ	68(38/30)	56.23 ± 3.27	OZ	68(36/32)	57.02 ± 3.19	⑨
Lan Pei Li 2012	HA+OZ	53(21/32)	68.59 ± 8.6	OZ	40(14/26)	66.33 ± 7.8	②+①
Lu Bin 2018	HA+OZ	41(23/18)	59.31 ± 9.37	HA	41(25/16)	58.26 ± 9.61	②+④
					41(19/23)	59.91 ± 9.85	
Cai Lixin 2018	OZ	30(16/14)	66.35 ± 6.05	HA	30(14/16)	66.42 ± 6.34	⑨
Ke Xinru 2019	HA	43(27/16)	65.12 ± 3.12	HA+OZ	43(29/14)	65.83 ± 3.59	②+①
Chen Xiaolong 2013	HA+OZ	60(34/26)	67.15 ± 6.24	HA	60(33/27)	67.48 ± 6.14	②
Huang Kaihua 2019	HA	37(8/29)	63.16 ± 7.12	PRP	33(8/25)	65.03 ± 7.10	⑦
				PRP+HA	31(8/23)	63 ± 7.02	
Liu Buyun 2017	PRP	26(6/12)	59.46 ± 5.93	HA	34(8/16)	61.76 ± 6.76	①+②
Machi Piao 2018	PRP	40(25/15)	58 ± 8	HA	40(24/16)	59 ± 9	②+①
Su xianLiang 2020	PRP	30(12/18)	49.02 ± 6.22	HA	30(14/16)	50.01 ± 5.89	⑥+③+②+④
Wang Guan Yu 2013	HA+OZ	126(34/92)	56.1 ± 13.7	HA	126(44/82)	51.1 ± 16.7	②
Xu Bujing 2015	PRP	20(6/14)	51.5 ± 12.9	HA	20(7/13)	52.5 ± 11.9	①
				PRP+HA	20(5/15)	52.5 ± 13.0	
Cao Jing 2020	PRP	50(17/33)	58.09 ± 3.72	HA	50(18/32)	58.13 ± 3.75	⑤
Fu Ligong 2017	PRP	33(17/16)	46.9 ± 7.5	HA	34(19/15)	47.2 ± 7.1	①
Xiang tai 2020	PRP	38(17/21)	75.01 ± 2.39	HA	38(20/18)	74.39 ± 1.25	⑩
Wang Changzheng 2017	OZ	100(44/56)	56.44 ± 5.89	HA	100(42/58)	58.01 ± 6.79	②+①
Sun Shaoyong 2018	HA+OZ	82(48/34)	59.01 ± 7.81	HA	61(32/29)	58.84 ± 8.02	②+①
Li Ming 2019	HA+OZ	40(25/15)	59.1 ± 9.9	HA	40 (12/28)	58.4 ± 10.3	④+③
Chen Yifei 2020	PRP	40 (20/20)	52.5 ± 2.0	HA	40(25/15)	52.3 ± 2.0	①
Yu Quan 2017	HA+0Z	41(19/22)	56.7 ± 7.3	HA	41(17/24)	55.9 ± 6.8	⑩

### 3.3. Methodological quality assessment of the included studies

Two investigators screened the literature according to the nadir criteria, excluded the literature that did not meet the inclusion criteria, then read the full text in detail, screened the eligible literature, cross-checked, and added a third investigator to discuss the decision if there was disagreement. The quality of the included literature was evaluated with reference to the Cochrane Handbook for Systematic Evaluation of the Cochrane Collaboration Network (version 5.1.0), which includes: the generation of randomized sequences; allocation concealment of randomized cases, implementation bias; blinded detection of study subjects and intervention implementers; blinded missing visit bias for outcome measures; completeness of data on outcome indicators; selective reporting of studies outcomes; other sources of bias.

### 3.4. Results of the mesh meta-analysis

#### 3.4.1. Evidence network diagram.

**Figure 2.**
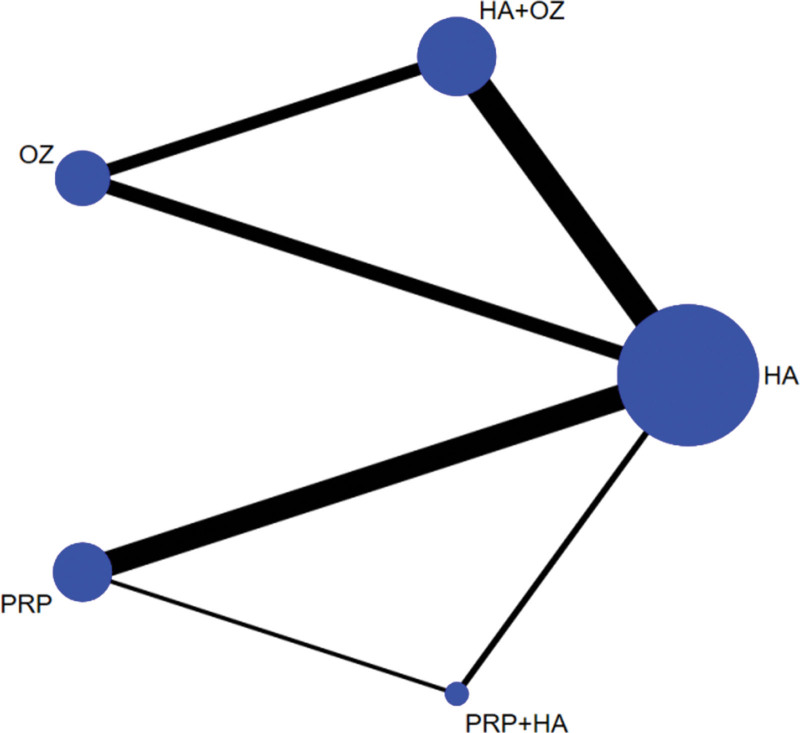
Evidence network diagram for the effectiveness of PRP, ozone, and sodium vitrate intra-articular injections for osteoarthritis of the knee.

Five treatment measures, 10 different two-by-two comparisons could be formed. A total of 8 direct comparisons exist for the 33 included studies (hyaluronic acid-hyaluronic acid+ozone, hyaluronic acid-platelet-rich plasma, hyaluronic acid-platelet-rich plasma+hyaluronic acid, hyaluronic acid-ozone, hyaluronic acid+ozoneplatelet-rich plasma, hyaluronic acid+ozone-ozone, platelet-rich plasma-hyaluronic acid, platelet-rich plasma+hyaluronic acid-ozone), and no direct research evidence exists for the remaining 2 comparisons, whose efficacy comparison results will be generated by indirect comparisons from the reticulated Meta-analysis. Figure 2 shows the network diagram of the evidence for the 33 included 5 treatment measures. In the figure, there are connecting lines between points indicating direct comparative evidence for the 2 interventions and no connecting lines indicating no direct comparative evidence.

#### 3.4.2. Contribution of the five interventions to the results of the web meta-analysis.

**Figure 3.**
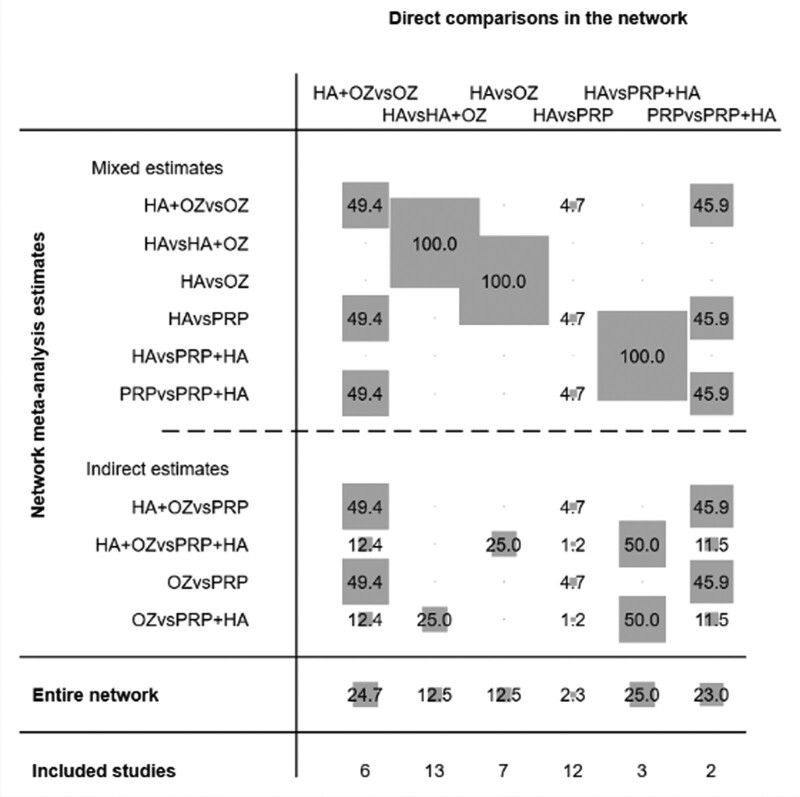
Contribution of the results of the net meta-analysis of the 5 interventions.

For further analysis of the impact and contribution of each direct comparison to the network meta-analysis, the value of the contribution of each group of direct comparisons to the study is expressed as gray circles and weight scores, Figure 3 shows the impact of different direct comparisons on the results of the mesh meta-analysis and the results of the whole network mesh meta-analysis in this study, the results of which suggest that for the whole network meta-analysis, hyaluronic acid vs. platelet-rich plasma+hyaluronic acid control direct comparison had the highest contribution (25.0%), followed by hyaluronic acid+ozone vs. ozone direct comparison (24.7%), platelet-rich plasma vs. platelet-rich plasma+hyaluronic acid direct comparison (23.0%), hyaluronic acid vs. both hyaluronic acid+ozone control and hyaluronic acid vs. ozone control direct comparison (12.5%), and hyaluronic acid vs. platelet-rich plasma direct comparison (2.3%).

#### 3.4.3. Inconsistency test.

**Figure 4.**
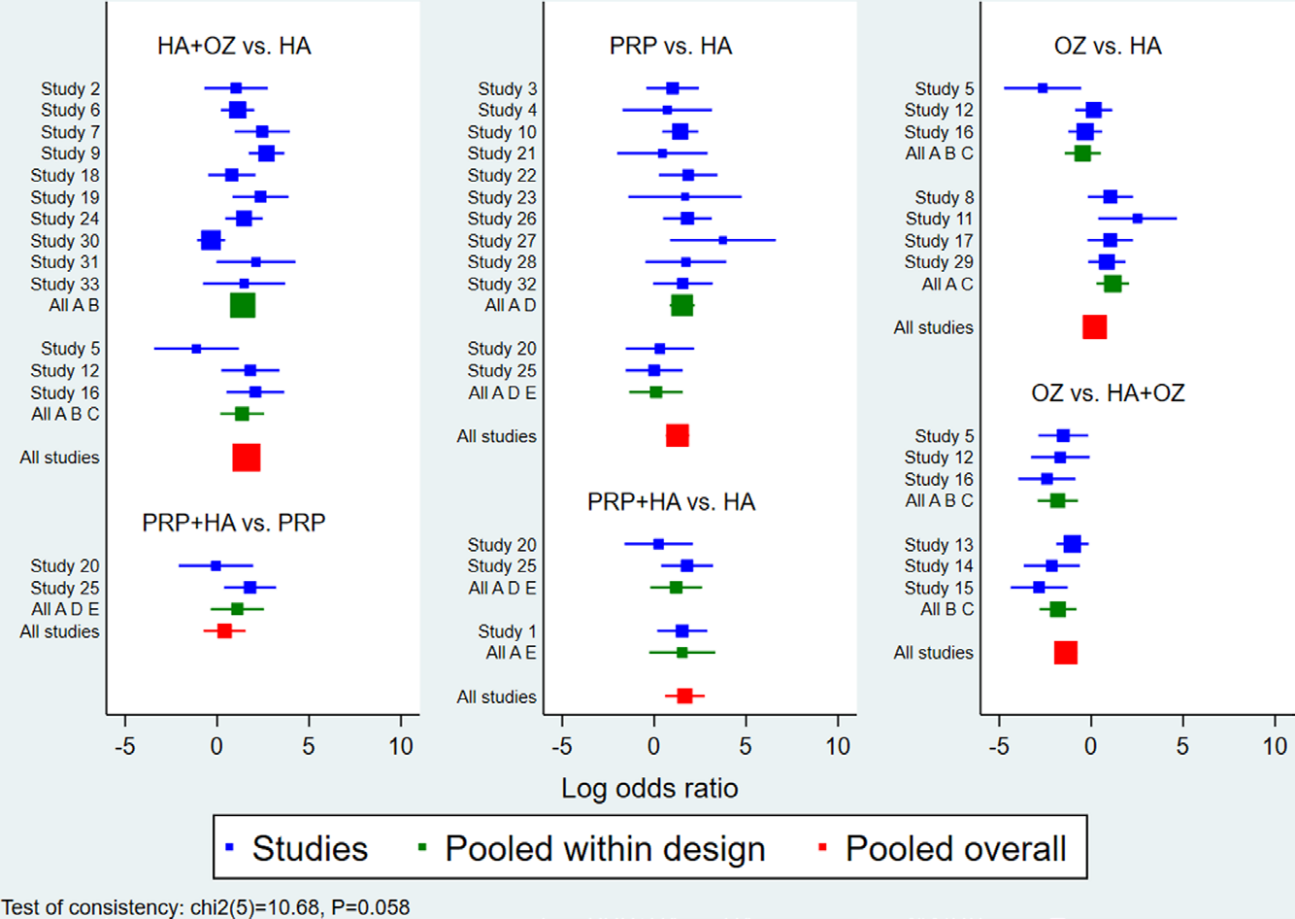
Results of inconsistency test for total efficiency.

**Figure 5.**
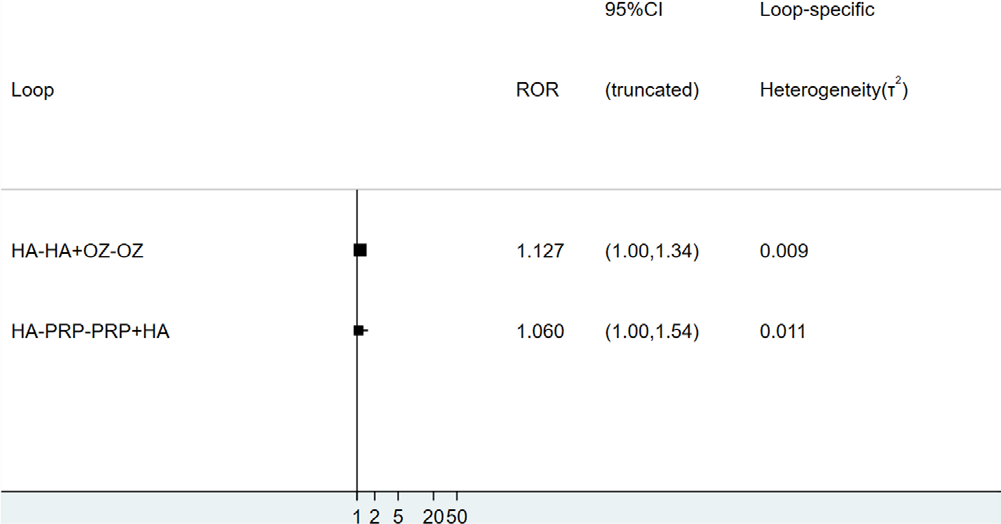
Results of inconsistency test between direct comparison and indirect comparison of total efficiency.

The global inconsistency test suggested *P* = .058>.05, indicating good consistency, as detailed in Figure 4; 2 trilateral rings (hyaluronic acid-hyaluronic acid+ozone-ozone, hyaluronic acid-platelet-rich plasma-platelet-rich plasma+hyaluronic acid). The consistency of the findings of each closed-loop study was tested, and the results showed that the inconsistency factor ROR was bounded by 1.060–1.127, and the lower limits of 95% CI were both 0.009 and 0.011, as detailed in Figure 5, which in summary indicates that the formation between the treatment measures of this study indicates that the consistency of each closed-loop is good.

#### 3.4.4. Small sample effect detection.

**Figure 6.**
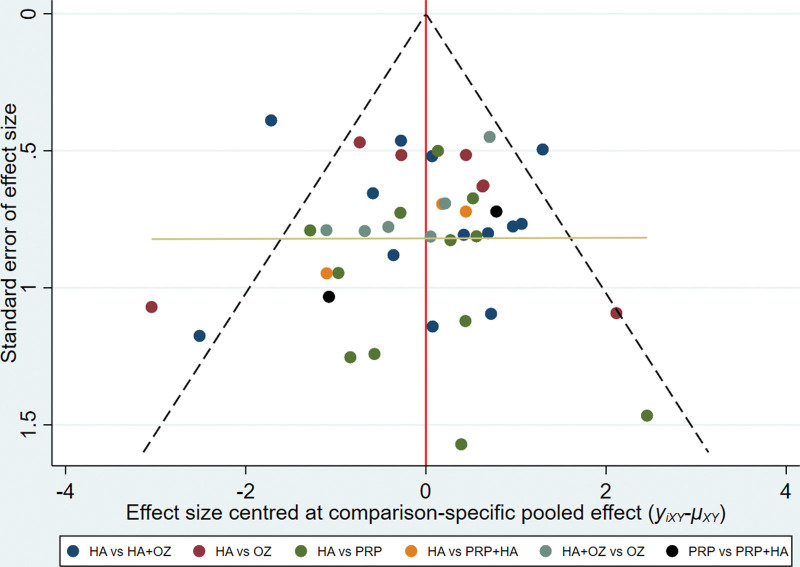
Total efficiency comparison of different interventions-correct funnel maps.

A comparison-corrected funnel plot was made for 5 of the 33 interventions included in this study. Different colored points in this funnel plot indicate different direct two-by-two comparisons, and the number of the same colored points indicates the number of that two-by-two comparison in the original study. If the funnel plot is symmetrical, it indicates that there is no significant small sample effect or publication bias. From Figure 6, it can be seen that the funnel plot is basically symmetrical, indicating that there is little possibility of a small sample effect or publication bias in the study.

#### 3.4.5. Results of reticulated meta-analysis.

The results of the random-effects model of MCMC based on Bayesian theory showed that Platelet-rich plasma was more effective than hyaluronic acid and ozone in monotherapy and the difference was statistically significant [platelet-rich plasma vs ozone: OR = 2.89, 95% CI (1.21, 6.88); platelet-rich plasma vs. ozone efficacy was not statistically significant compared to hyaluronic acid [OR = 1.22 95% CI (0.68, 2.21).

Comparison of combination therapy with monotherapy: the efficacy of the 2 included combination therapies (platelet-rich plasma+hyaluronic acid, hyaluronic acid+ozone) was compared with 3 monotherapies (platelet-rich plasma, ozone, hyaluronic acid), with the exception of hyaluronic acid+ozone and platelet-rich plasma+hyaluronic acid vs. platelet-rich plasma where the difference in efficacy was not statistically significant (hyaluronic acid+ozone vs. platelet-rich plasma: OR = 1.38, 95% CI (0.62, 3.10); platelet-rich plasma+ hyaluronic acid vs. platelet-rich plasma=OR = 1.50, 95% CI (0.48, 4.70)), the rest were statistically significant (platelet-rich plasma+hyaluronic acid vs. ozone: OR = 4.34, 95% CI (1.27, 14.84), platelet-rich plasma+hyaluronic acid vs. hyaluronic acid: OR = 5.31, 95% CI (1.81, 15.62), hyaluronic acid+ozone vs. ozone: OR = 4.00, 95% CI (2.15, 7.44), hyaluronic acid+ozone vs. hyaluronic acid: OR = 4.89, 95% CI (2.97, 8.06)), indicating that this combined platelet-rich plasma+hyaluronic acid and hyaluronic acid+ozone therapy was superior to single ozone and hyaluronic acid treatment. The comparative results of the efficacy of each treatment measure for osteoarthritis of the knee are shown in Table 2.

**Table 2:** Network meta-analysis results of the efficacy for knee osteoarthritis in different knee cavity injections treatments.

PRP+HA	0.92 (0.28,3.03)	0.67 (0.21,2.09)	0.23 (0.07,0.79)	0.19 (0.06,0.55)
1.09 (0.33, 3.57)	HA+OZ	0.72 (0.32,1.62)	0.25 (0.13,0.47)	0.20 (0.12,0.34)
1.50 (0.48, 4.70)	1.38 (0.63,3.10)	PRP	0.35 (0.15,0.82)	0.28 (0.15,0.53)
4.34 (1.27, 14.84)	4.00 (2.15,7.44)	2.89 (1.21,6.88)	OZ	0.82 (0.45,1.47)
5.31 (1.8, 15.62)	4.89 (2.97,8.06)	3.54 (1.88,6.68)	1.22 (0.68,2.21)	HA

Table Note: The data in the following cells of the code represent the corresponding columns: OR (95% CI) for comparison of the efficacy of row treatment measures; 95% CI does not contain 1 indicates statistical significance, OR value greater than 1 indicates that the efficacy of the column treatment measures is better than that of the row treatment measures; OR value less than 1 indicates that the efficacy of the column treatment measures is inferior to that of the row treatment measures; 95% CI contains 1 indicates no statistical significance, and the efficacy of the 2 treatment measures cannot yet be considered different.

#### 3.4.6. Reticulated meta-ranking results of the total effective rate.

**Figure 7.**
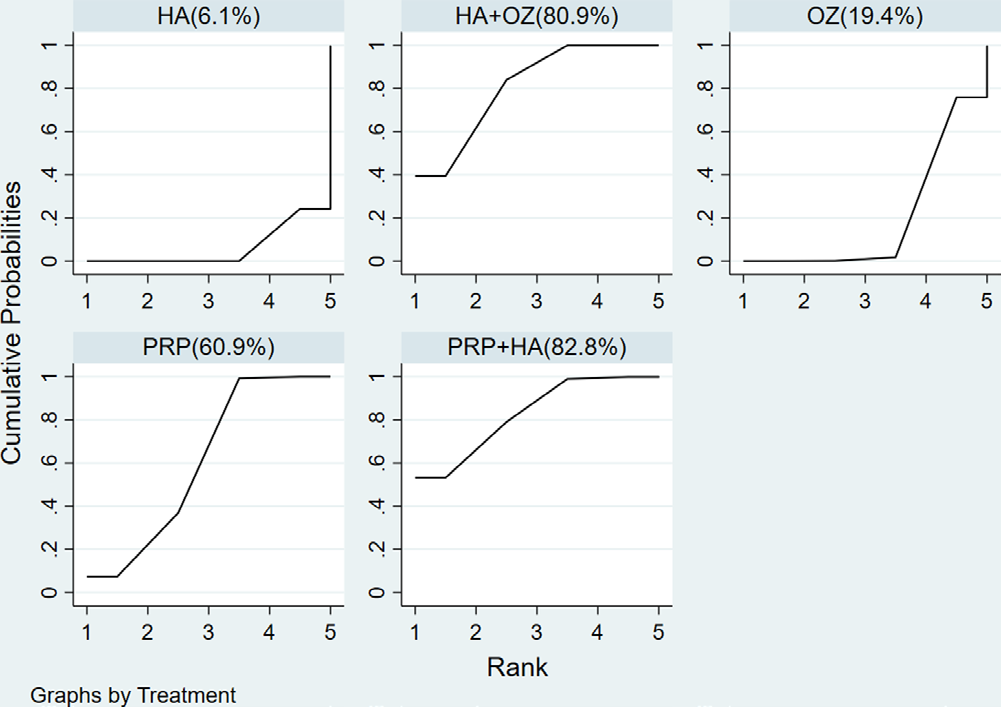
Plots for the surface under the cumulative ranking curves of 5 interventional strategies for the treatment of knee osteoarthritis.

Note: Before the figure is the treatment measure name in parentheses, and the number in parentheses indicates the area under the SUCRA curve. The larger the area, the better the treatment effect.

According to the MCMC method random effect model comparison results, the SUCRA curve was plotted and the area under the curve was used to predict the ranking of efficacy, see Figure 7. The results also showed that platelet-rich plasma+hyaluronic acid and hyaluronic acid+ozone were better than monotherapy and platelet-rich plasma was better than hyaluronic acid and ozone in monotherapy.

#### 3.4.7. Sensitivity analysis.

The data were statistically analyzed again using the MCMC method fixed-effects model and the SUCRA graphs were plotted with the same ranking results as the random-effects model, indicating that the results of this study are more stable.

## 4. Discussion

Arthritis cavity injection therapy is a commonly used conservative treatment for knee osteoarthritis, and the commonly used joint cavity injection drugs are mainly platelet-rich plasma, ozone injection solution, and sodium hyaluronate. The existing original studies are mostly single clinical efficacy analysis of platelet-rich, ozone, and sodium hyaluronate, and there is a lack of direct comparison of comprehensive therapy between platelet-rich, ozone, and sodium hyaluronate and their combination. This prevents researchers from comprehensively and systematically evaluating the clinical efficacy of platelet-rich, ozone, and sodium hyaluronate joint cavity injections, which is not conducive to the selection and promotion of optimal treatment regimens. In this study, a mesh meta-analysis was performed in the field of knee joint cavity injections to compare the differences in efficacy between platelet-rich, ozone, and sodium hyaluronate combined therapies and between their combinations, solving the problem of lack of direct comparison of joint cavity injection therapies in the original study, which made it difficult to determine the differences in efficacy of different knee joint cavity injections. Therefore, the authors’ current study provides more reliable evidence-based medicine for a more comprehensive understanding and objective evaluation of combination therapies between platelet-rich, ozone, and sodium hyaluronate, as well as combinations between them. Thirty-3 RCTs comparing between platelet-rich, ozone, and sodium hyaluronate and between combinations of these therapies were included in the database, with a combined sample size of 7003 cases and 5 therapeutic measures. The results showed that platelet-rich plasma+hyaluronic acid versus hyaluronic acid+ozone combination therapy was superior to single ozone injection and hyaluronic acid injection therapy, and that platelet-rich plasma was superior to ozone injection and hyaluronic acid in monotherapy.

Most studies now consider knee cartilage damage, reduced synovial fluid in the joint cavity and aseptic inflammation as one of the main causes of clinical symptoms of knee osteoarthritis.^[44]^ Platelet-rich-plasma contains a large amount of growth factors and has a role in repairing articular cartilage in osteoarthritis of the knee.^[45]^ Hyaluronic acid is the main component of synovial fluid, which acts as a lubricant in the joint cavity, reduces cartilage wear, and has a protective effect on articular cartilage.^[46]^ In contrast, ozone belongs to a strong oxidant with strong anti-inflammatory, analgesic and cartilage repair effects, and has some efficacy in the treatment of osteoarthritis of the knee joint.^[47]^ However, among these monotherapies, there was no significant difference in the efficacy of ozone injection compared with hyaluronic acid, and the efficacy of platelet-rich plasma injection was superior to both ozone and hyaluronic acid therapies. However, both hyaluronic acid+ozone and platelet-rich plasma+hyaluronic acid efficacy were superior to ozone and hyaluronic acid monotherapies. Therefore, it is believed that in the course of clinical practice, hyaluronic acid+ozone or platelet-rich plasma+hyaluronic acid combination therapy or platelet-rich plasma therapy can be preferred for Kellgren-Lawrence grade II-III patients in combination with their conditions.

The lack of direct comparisons with the included platelet-rich plasma+ozone and steroid hormone treatments affects the comprehensiveness of the evaluation of the efficacy of knee joint cavity injections. In practical clinical use, these treatments may have better efficacy than the therapeutic measures included in this study. Therefore, more comprehensive clinical studies are necessary to provide more reliable evidence for knee cavity injections in the treatment of knee osteoarthritis. However, no significant asymmetry was found in the study comparison-corrected funnel plot, suggesting no significant small sample effect or publication bias, inconsistency tests suggesting good consistency across closed loops, and random-effects model efficacy ranking in the sensitivity analysis was the same as the fixed-effects model, indicating stable findings for these 5 joint cavity injection treatment measures.

In summary, reticulated meta-analysis provided a more systematic and objective evaluation of the effectiveness of different joint cavity injection therapies in the treatment of knee osteoarthritis, providing a more comprehensive and clearer understanding of different joint cavity injection therapies and facilitating the selection of the best treatment plan for knee osteoarthritis. The results concluded that among the 5 knee joint cavity injection therapies included, hyaluronic acid+ozone and platelet-rich plasma+hyaluronic acid were more effective than ozone and hyaluronic acid monotherapy, while platelet-rich plasma was more effective than hyaluronic acid and ozone. in practice, hyaluronic acid+ozone and platelet-rich plasma+hyaluronic acid combination therapy or platelet-rich plasma therapy can be preferred based on the findings of this study combined with physicians’ own treatment experience. Based on the shortcomings of the existing studies, the findings of this study still need to be confirmed by a large number of well-designed and appropriate RCTs covering a variety of Chinese medical methods.

## Author contributions

Conceptualization: Fang Zhi, Xingzhen Lin.

Data curation: Xinju Hou,Xiongwei Deng.

Formal analysis: Qin Lan, Xingzhen Lin.

Investigation: Xingzhen Lin.Fang Zhi.

Resources: Xingzhen Lin..Fang Zhi.

Software: Xingzhen Lin.Qin Lan.

Supervision: Fang Zhi, Xiongwei Deng.

Writing – original draft: Xingzhen Lin, Xinju Hou.

Writing – review & editing: Xingzhen Lin, Qing Wan.
